# City life makes females fussy: sex differences in habitat use of temperate bats in urban areas

**DOI:** 10.1098/rsos.140200

**Published:** 2014-11-19

**Authors:** Paul R. Lintott, Nils Bunnefeld, Elisa Fuentes-Montemayor, Jeroen Minderman, Rebekah J. Mayhew, Lena Olley, Kirsty J. Park

**Affiliations:** Biological and Environmental Sciences, School of Natural Sciences, University of Stirling, Stirling FK9 4LA, UK

**Keywords:** sex differences, habitat use, urbanization, fragmented woodland, bats

## Abstract

Urbanization is a major driver of the global loss of biodiversity; to mitigate its adverse effects, it is essential to understand what drives species' patterns of habitat use within the urban matrix. While many animal species are known to exhibit sex differences in habitat use, adaptability to the urban landscape is commonly examined at the species level, without consideration of intraspecific differences. The high energetic demands of pregnancy and lactation in female mammals can lead to sexual differences in habitat use, but little is known of how this might affect their response to urbanization. We predicted that female *Pipistrellus pygmaeus* would show greater selectivity of forging locations within urban woodland in comparison to males at both a local and landscape scale. In line with these predictions, we found there was a lower probability of finding females within woodlands which were poorly connected, highly cluttered, with a higher edge : interior ratio and fewer mature trees. By contrast, habitat quality and the composition of the surrounding landscape were less of a limiting factor in determining male distributions. These results indicate strong sexual differences in the habitat use of fragmented urban woodland, and this has important implications for our understanding of the adaptability of bats and mammals more generally to urbanization.

## 2. Introduction

Urbanization is driving the fragmentation of landscapes at an unprecedented rate and is therefore a significant contributing factor to the current biodiversity crisis [1]. Understanding patterns of habitat use and its drivers within the urban matrix is crucial to minimize its adverse effect on biodiversity [2], taking into account the impact of urbanization at a variety of spatial scales [3]. While many studies of urban biodiversity have focused on species-level responses, there has been relatively little consideration of the potential importance of intraspecific differences.

Sexual differences in animal behaviour and habitat use is taxonomically widespread and one of the most commonly studied concepts in biology, identified and investigated as far back as Darwin [4]. Habitat segregation between sexes can occur because of differences in antipredation behaviour during the breeding period (e.g. Eurasian wild sheep [5]), differences in social motivation to interact that may lead to behavioural incompatibility (e.g. red deer [6]), physiological differences (e.g. pelagic shark [7]), or to decrease intraspecific resource competition (e.g. New Zealand sea lion [8]). These behaviours often result in segregation between distinct habitat types; however, we have relatively little information about whether similar patterns occur within urban landscapes.

There are few other orders of animals that are as strongly associated with people as bats. Human habitations provide roosts, while adaptations of the environment supply food sources, such as insects at artificial light sources [9]. However, while many species have adapted to exploit the urban landscape, the general pattern is of declining bat activity and bat species richness with increasing levels of urbanization [10,11].

The species diversity, variety of social systems and tendency among some species to segregate during the maternity season make bats an ideal taxon for studying sex differences in habitat use; however, relatively little attention has been paid to this subject [12]. Sexual segregation may occur within the roost [12], while foraging [13], and during migration [14]. The energetic demands of pregnancy and lactation can limit females to foraging within highest quality habitats, thereby excluding them from marginal upland habitat [15] and arable land [16]. Conversely, habitat quality is less of a limiting factor for males and non-breeding females as they have lower energy demands and are able to use torpor more frequently during the summer to maximize energy savings [12].

Woodland is widely regarded as a primary habitat for bats [17], however, within the urban matrix it is of variable quality, subject to invasive species encroachment and often consists of small, fragmented patches [18]. Consequently, the vegetation characteristics of urban woodland influence bat species presence and community composition [19]. Differences in habitat requirements between males and females may further limit the distribution of a species within the urban matrix but there is little known about the potential for sexual differences as most studies of bats in urban environments are conducted using acoustic detectors which are unable to distinguish between sexes.

We hypothesize that at the local scale, the variable quality of urban woodland may limit females as they are frequently restricted to foraging within high-quality habitats. Additionally, the necessity of females to commute between foraging and roosting locations owing to the demands of lactation will make the composition, spatial configuration and heterogeneity of the landscape surrounding woodland relatively more important for females than males. Thus, we predict that female *Pipistrellus pygmaeus* will show greater selectivity of foraging locations within fragmented urban woodland in comparison to males, and that this difference will be expressed at both a local and landscape level.

In this paper, we therefore use 128 h of trapping data to test whether male and female *P. pygmaeus*, a widespread species within Europe, differ in their use of fragmented urban woodland. Rather than examining broad-scale differences in use between urban and non-urban habitat, we are testing how differences in habitat characteristics at a fine spatial scale, and the composition of the surrounding matrix, may lead to sex differences in habitat use within the urban landscape.

## 3. Material and methods

### 3.1 Site selection

We identified 32 urban woodland study sites in central Scotland (appendix A; figure 2) using Ordnance Survey digital maps [20], which we surveyed between 19 May 2011 and 1 September 2011. Urban areas were designated as those where urban cover was the dominant land use within a 1 km grid square (i.e. the proportion of the grid square containing urban grey space was greater than all alternative habitat types) as categorized by the Centre for Ecology and Hydrology Land Cover Map 2000 (LCM2000). Sites were selected by size, longitude and degree of urbanization in the surrounding 1 km using a stratified random sampling method. Selected woodlands were a minimum of 50 years old and were either broadleaved or consisted of a mixture of conifer and broadleaved trees. We surveyed sites in random order through the field season to avoid any spatial or temporal bias.

### 3.2 Vegetation surveys

We conducted daytime vegetation surveys within a week of the bat survey to ensure that appropriate vegetative conditions were recorded. Four circular plots with radii of 20 m were randomly located within each woodland patch. At each of the four plots, all trees were counted, identified to at least genus level and tree basal area measured. Vegetation clutter was measured from 0 to 4 m in height at 18 evenly spaced points within each plot to determine vertical forest structure; adopting a similar approach to Smith & Gehrt [19], a 4 m pole with sixteen 0.25 subsections marked upon it was placed at each point within the plot. Any foliage, branches or stems touching a subsection was counted and summed to provide a measure of clutter (100% clutter occurred when foliage touched all points on the pole at every point within the plot). Within each plot, canopy cover (%) was assessed at 18 points in each plot using a sighting tube with an internal crosshair; if the crosshair intersected canopy vegetation, presence of canopy was recorded [21]. Data for the four vegetation plots were combined to provide a description of each woodland patch. Additionally, we visually assessed the remaining woodland to ensure that the vegetation surveys were representative of the entire woodland patch.

### 3.3 Bat surveys

We used one Austbat harp trap (2.4×1.8 m) and three Ecotone mist nets (2.4×6 m each) within each woodland to provide an estimate of the relative abundance of male and female *P. pygmaeus*. A trap was placed in each of the plots that had previously been surveyed for vegetation. An acoustic lure was used to increase trapping rate (as described by Lintott [22]). We commenced trapping 30 min after sunset to avoid the peak emergence and commuting time for *P. pygmaeus*. Traps were checked every 15 min to extract any captured bats, which were then identified to species, aged, sexed, measured, weighed and marked temporarily by fur clipping.

### 3.4 Landscape analysis

We plotted bat trap locations using ArcGIS 10 [23] and determined the centre point of the four traps within each site. Buffers of 250, 500, 1000, 1500 and 2000 m radius were created around the central point reflecting the upper limit of home range size for *P. pygmaeus* [24]. Data from the OS MasterMap Topography Layer [20] was used to reclassify the landscape within each buffer into a set of discrete biotope types. These were: (i) greyspace (buildings, structures, roads and paths); (ii) green space (gardens, parkland, managed grassland, rough grassland and farmland); (iii) inland fresh water; and (iv) woodland (coniferous, deciduous and mixed woodland). Woodland Euclidean nearest neighbour distance (ENN, the mean value of ENN distances between all woodland patches within the landscape) and the Shannon diversity index (SHDI, a measure of landscape heterogeneity) were calculated as previous studies have found these variables to be important [25]. The proportion of land covered by each biotope, woodland ENN and SHDI were calculated for each buffer scale using Fragstats v. 4.0 [26].

### 3.5 Data analysis

We undertook statistical analyses using R v. 2.14 [27] using the lme4 [28] and effects package [29]. We performed a general linear mixed-effects model (GLMMs) with binomial error distribution and a logit link to quantify the influence of woodland characteristics and landscape metrics on male and female abundance. In order to assess the relative effects of these variables on males in comparison to females, the model was run with the proportion of females to males per trap (*n*=128) as the response variable, with ‘site’ included as a random (grouping) factor. Based upon the scientific literature on the ecology of woodland bats [25] the following predictor variables were included in the model: (i) woodland vegetation characteristics: tree species richness, average tree basal area, woodland clutter and woodland canopy cover (covariates) and woodland type as a fixed factor; (ii) patch configuration: woodland size, woodland shape (covariates) and the interaction between size and shape. (Woodland shape is the perimeter divided by the minimum perimeter possible for a maximally compact patch of the same area. This equals 1 when the patch is maximally compact and increases as shape becomes irregular [26].); and (iii) landscape metrics (covariates). Temperature and date were also included in all models as covariates. We assessed landscape metrics for issues of multicollinearity, and used GLMMs for abundance with single landscape parameters (at each spatial scale) as a preliminary assessment of which key landscape predictors should be included in the final model.

All resulting predictor variables were tested for collinearity, however, none were considered to be strongly correlated based upon a Pearson correlation coefficient of greater than or equal to 0.6 and *p*≤0.05. Continuous predictor variables were centred and standardized following Schielzeth [30] to allow direct comparison of the size of estimated coefficients. We present the result of the full model including standardized parameters and confidence intervals for all explanatory variables. Inferences on the effect of each parameter were made by: (i) comparing its standardized estimate with other predictor variables to determine relative importance; (ii) the upper and lower 95% quantiles of each parameter distribution obtained from *n*=2000 simulated draws from the estimated distribution [31]; and (iii) a comparison of models excluding each parameter in turn using likelihood ratio tests (LRTs [32]). LRTs of main effect parameters also involved in interactions were performed by comparing the model excluding the main effect term to the model including all main effects (but not interactions) only. Prediction plots were constructed by undertaking simulated draws (*n*=2000) from the estimated distribution of one explanatory variable while maintaining all other parameters in the model at their mean observed values.

## 4. Results

We captured 162 *P. pygmaeus* within 27 of the 32 woodlands. The sample population comprised 67 adult males (41%) within 25 woodlands and 55 adult females (34%), 52 of which were classified as breeding females, within 19 woodlands. We caught the first juvenile on 10 July and from this date onwards, 40 juveniles (25%) were captured in 12 of the 23 woodlands surveyed. Juveniles were found in an insufficient number of sites were therefore excluded from further analysis.

**Figure 1.**
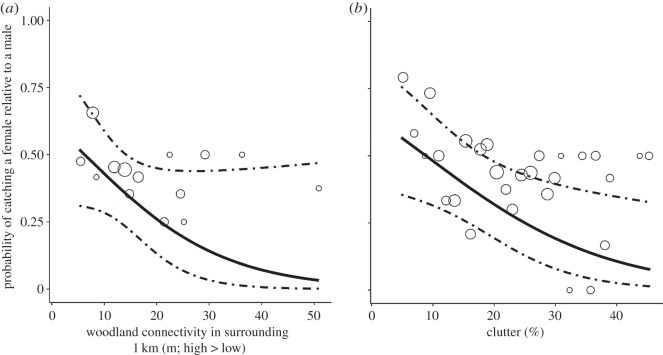
Estimated probability of finding a female relative to a male *P. pygmaeus* in fragmented urban woodland. Dashed lines indicate 95% confidence intervals. Original data on the proportion of females are superimposed as grey circles with diameter proportion to the total number of females. Woodland connectivity (*a*) is measured using the Euclidean nearest neighbour distance (ENN, the mean value of ENN distances between all woodland patches within the landscape). A landscape containing highly connected woodlands would have a low ENN value, while poorly connected woodlands would have a high ENN value.

**Figure 2.**
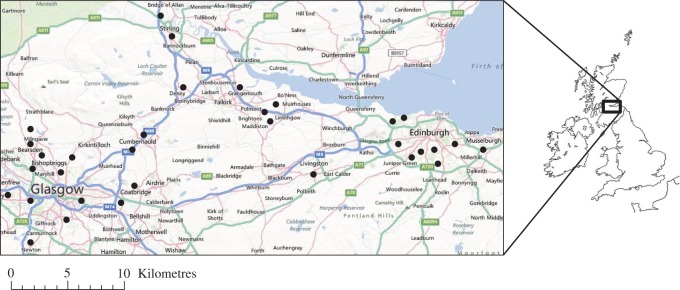
Map of central Scotland showing approximate locations of woodland sites (black dots) surveyed in 2011.

The importance of woodland vegetation characteristics, patch configuration and the surrounding landscape differed between the sexes (table 1). Woodland isolation (ENN) in the surrounding 1 km had the largest effect size and a negative influence on the probability of capturing a female. Based on the estimated coefficients in table 1, the predicted probability of capturing a female was 0.03 (0.002–0.36) in isolated woodland, 0.24 (0.14–0.39) in moderately connected woodland, while there was little difference in the probability of finding either males (0.52; 0.28–0.75) or females (0.48; 0.25–0.72) in well-connected woodland (figure 1*a*). Similarly, while there was a similar likelihood of capturing either males (0.42; 0.22–0.64) or females (0.58; 0.36–0.78) in woodlands with low (5%) woodland clutter, females avoided highly cluttered locations; the probability of finding a female in woodland containing 45% clutter was 0.08 (0.02–0.32; figure 1*b*). Woodland shape and average tree basal area were both marginally significant predictors of sex differences in habitat use. There were similar probabilities of capturing either females (0.42; 0.24–0.63) or males (0.58; 0.37–0.76) in compact woodland, however, this contrasted with complex woodland with a high edge to interior ratio where the probability of capturing a female was much lower at 0.14 (0.03–0.46). The probability of capturing a female increased in woodland with a high tree basal area. An increase in average tree basal area from 10 to 40 cm^2^ led to an increase in the probability of capturing a female from 0.39 (0.26–0.55) to 0.8 (0.24–0.98), while declining for males from 0.61 (0.45–0.74) to 0.2 (0.02–0.76). Additionally, the probability of capturing a female was increased in woodlands with well-connected urban waterways in the surrounding 1 km, however, the effect size was relatively small (table 1).

**Table 1.** Parameter estimates and likelihood ratio tests of the GLMM for the relative proportion of the number of trapped female *P. pygmaeus* to males in urban woodland. (The model was run to calculate the probability of finding a female relative to a male; hence positive estimates refer to an explanatory variable that relates to an increased probability of finding a female. The most important landscape parameters at the most important spatial scale for either sex were included in the model. Test statistics derived from the deletion of each term from the full model (for the two-way interaction) and from the model with main effects only (main effect terms).)

fixed effects	estimate (±s.e.)	log likelihood	*χ*^2^	*χ*^2^ d.f.	*p*
intercept	−0.58±0.40				
date	0.07±0.30	−31.49	0.05	1	0.83
temperature	0.14±0.32	−31.54	0.16	1	0.69
tree basal area	0.50±0.31	−33.11	3.30	1	0.07
tree species richness	0.42±0.28	−32.67	2.42	1	0.12
woodland canopy cover	−0.39±0.29	−32.35	1.78	1	0.18
woodland clutter	−0.64±0.26	−34.95	6.97	1	0.01
woodland shape	−0.50±0.30	−32.97	3.02	1	0.08
woodland size	−0.13±0.26	−31.57	0.21	1	0.65
woodland type	−0.84±0.67	−32.28	1.63	1	0.2
water connectivity (1 km)	−0.13±0.22	−33.26	3.60	1	0.06
woodland connectivity (1 km)	−0.87±0.44	−33.77	4.61	1	0.03
shape × size	0.13±0.44	−33.03	3.22	2	0.36

## 5. Discussion

This study demonstrates the importance of habitat quality and connectivity to breeding female bats in the built-up landscape and has important implications for our understanding of the adaptability of this species to human disturbed landscapes. Lower abundance of *P. pygmaeus* females within poorly connected woodland patches of complex shapes with high clutter levels and small average tree basal areas suggests that differences in habitat use between the sexes occur not only at a broad, between-habitat scale [15] but also within habitats, at a fine spatial scale.

Male and female *P. pygmaeus* demonstrated marked differences in their response to the character of fragmented urban woodland. The lack of selectivity exhibited by males suggests that they are able to use a wider range of conditions as they have lower energy demands than reproductive females [33]. Females face higher energetic demands during pregnancy and lactation, and have a relatively shorter time period to accumulate sufficient fat for the following hibernation period [13]. Additionally, reproductive females use torpor less frequently than males as it can reduce fetal growth rates [34]. Female response to the vegetation characteristics and patch configuration of urban woodland is therefore likely to reflect selective foraging in optimum habitats. The wing shape and echolocation call of *P. pygmaeus* makes it well adapted for foraging in open habitats [35], which appears to be demonstrated in female preference for woodlands containing reduced woodland clutter. The association between female abundance and large average tree basal areas, a trait associated with mature woodlands [36], may occur as woodlands containing larger trees can provide a larger number of microhabitats and therefore more foraging opportunities, a greater availability of night roosts [37], and reduced predation risk [38]. Higher female abundance within compact woodland suggests a preference for woodland patches which expose proportionally less edge to the surrounding urban matrix. Woodland edges in the urban matrix are often adjacent to habitats under high anthropogenic pressure and can often comprise only those tree species and invertebrate populations that are able to tolerate such conditions [39]. It may be that the combination of anthropogenic disturbance (i.e. noise or light pollution) and reduced prey availability provide poorer foraging habitat for females in contrast to woodland interior.

The relative importance of the landscape surrounding urban woodland for females may reflect the differences in roosting strategies between the sexes. The importance of woodland connectivity for females is probably driven by the necessity of lactating females to return frequently to the roost. Radio tracking of lactating *P. pygmaeus* females shows that, on average, they return to their roost 3.7 times per night [40]. While the roost sites of females captured during this study is unknown, the use of well-connected woodlands will reduce the necessity to commute across the urban matrix. This will decrease the perceived predation risk of commuting across open habitats alongside reducing the extent of anthropogenic disturbances (e.g. noise and light pollution or the risk of vehicle collisions [41,42]). The daily energy expenditure of reproductive females can double by peak lactation [43]; making it imperative that foraging flights are of optimal efficiency. Conversely, males are not constrained by the requirement to return to a particular roost during the night, often roosting either alone or in small groups in separate roosts [12].

We found no evidence of spatially separated habitat use between sexes, as males were just as likely to be found in those habitats preferentially selected by females. However our results suggest that intra-male segregation may be occurring; with males in poorer habitat potentially suffering reduced foraging efficiency which may have subsequent consequences for reproductive fitness or survival over winter [44]. The mechanism behind segregation is unknown, although suggestions from past studies have included females (and the males that share their roost) excluding other males from their home range [12] to differences in physiological and social needs [13]. Here we show that habitat quality appears to be less of a limiting factor for males who appear to make wider use of the urban matrix (i.e. poorly connected woodland) and can tolerate higher anthropogenic pressure (i.e. complex woodlands with more pronounced edge effects), which may be driving sex differences in habitat use. Late summer and autumnal activities such as mating behaviour may change habitat use in both sexes given that there will be a stronger pressure for males to frequent similar localities as females, and that females will be less restricted in habitat choice by not having to return to a maternity roost. Although we accounted for date in our model, the extent to which the differential habitat selection between the sexes continues into the mating period is unknown and future research on this would be of value.

The vulnerability of bat species to human disturbed landscapes is often assessed through use of acoustic surveys [45] which allow researchers to quantify relative levels of bat activity between habitats. For example, in urban environments foraging activity of *P. pygmaeus* is higher within the woodland interior than along woodland edge habitat, urban grey space and non-wooded green space [46]. There is evidence, at least for some species (including *P. pygmaeus*), that foraging activity recorded via acoustic surveys can be used as a surrogate for abundance without the need to trap, which can be a costly and time-consuming process which requires expertise [22]. However, our results highlight the value of trapping data which enables differences in habitat selection between males and females to be assessed, something which is not possible using acoustic monitoring. Acoustic surveys using bat detectors may therefore distort our perception of how tolerant bats are to anthropogenic disturbance. While trapping is a more intensive and intrusive survey technique, and necessarily limited to smaller geographical regions, studies such as these are important in complementing large-scale, long-term acoustic monitoring (e.g. National Bat Monitoring Programme; Bat Conservation Trust 2013) in identifying key habitats for breeding females and how to optimize their management. As urbanization continues to contribute to the global loss of biodiversity it is imperative that monitoring strategies are optimized to ensure that a true understanding of the scale of loss is gained. This study shows that determining species presence may not be a satisfactory indicator of adaptability or tolerance to the urban matrix if there are sexual differences in habitat selection.

## Data accessibility

Data available from the Dryad repository http://doi.org/10.5061/dryad.5p5dd.
